# A Novel Approach for Manual Segmentation of the Amygdala and Hippocampus in Neonate MRI

**DOI:** 10.3389/fnins.2019.01025

**Published:** 2019-09-24

**Authors:** Niloofar Hashempour, Jetro J. Tuulari, Harri Merisaari, Kristian Lidauer, Iiris Luukkonen, Jani Saunavaara, Riitta Parkkola, Tuire Lähdesmäki, Satu J. Lehtola, Maria Keskinen, John D. Lewis, Noora M. Scheinin, Linnea Karlsson, Hasse Karlsson

**Affiliations:** ^1^FinnBrain Birth Cohort Study, Turku Brain and Mind Center, Institute of Clinical Medicine, University of Turku, Turku, Finland; ^2^Department of Psychiatry, Turku University Hospital, University of Turku, Turku, Finland; ^3^Turku Collegium for Science and Medicine, University of Turku, Turku, Finland; ^4^Department of Biomedical Engineering, Case Western Reserve University, Cleveland, OH, United States; ^5^Department of Medical Physics, Turku University Hospital, Turku, Finland; ^6^Department of Radiology, Turku University Hospital, University of Turku, Turku, Finland; ^7^Department of Pediatric Neurology, Turku University Hospital, University of Turku, Turku, Finland; ^8^Montreal Neurological Institute, McGill University, Montreal, QC, Canada; ^9^Turku PET Centre, University of Turku, Turku, Finland; ^10^Department of Child Psychiatry, Turku University Hospital, University of Turku, Turku, Finland

**Keywords:** magnetic resonance imaging, manual segmentation, automated segmentation, infants, brain, amygdala, hippocampus

## Abstract

The gross anatomy of the infant brain at term is fairly similar to that of the adult brain, but structures are immature, and the brain undergoes rapid growth during the first 2 years of life. Neonate magnetic resonance (MR) images have different contrasts compared to adult images, and automated segmentation of brain magnetic resonance imaging (MRI) can thus be considered challenging as less software options are available. Despite this, most anatomical regions are identifiable and thus amenable to manual segmentation. In the current study, we developed a protocol for segmenting the amygdala and hippocampus in T2-weighted neonatal MR images. The participants were 31 healthy infants between 2 and 5 weeks of age. Intra-rater reliability was measured in 12 randomly selected MR images, where 6 MR images were segmented at 1-month intervals between the delineations, and another 6 MR images at 6-month intervals. The protocol was also tested by two independent raters in 20 randomly selected T2-weighted images, and finally with T1 images. Intraclass correlation coefficient (ICC) and Dice similarity coefficient (DSC) for intra-rater, inter-rater, and T1 vs. T2 comparisons were computed. Moreover, manual segmentations were compared to automated segmentations performed by iBEAT toolbox in 10 T2-weighted MR images. The intra-rater reliability was high ICC ≥ 0.91, DSC ≥ 0.89, the inter-rater reliabilities were satisfactory ICC ≥ 0.90, DSC ≥ 0.75 for hippocampus and DSC ≥ 0.52 for amygdalae. Segmentations for T1 vs. T2-weighted images showed high consistency ICC ≥ 0.90, DSC ≥ 0.74. The manual and iBEAT segmentations showed no agreement, DSC ≥ 0.39. In conclusion, there is a clear need to improve and develop the procedures for automated segmentation of infant brain MR images.

## Introduction

The study of infants’ brain structures provides us with the means to investigate the timing of the structural and functional development (Jernigan et al., 2011). In infants, magnetic resonance imaging (MRI) is a safe tool that aids the investigation of postnatal maturational changes, such as myelination, and how these changes relate to behavioral development (Jernigan et al., 2011; Devi et al., 2017).

Brain MRI segmentation is one of the most critical tasks in many clinical applications (Balafar et al., 2010). Segmentation of different tissue types from brain magnetic resonance (MR) images is an important step in studying and analyzing brain anatomy and, consequently, the dynamic processes that occur during development (Wang et al., 2012).

Despite the good availability of automated and semi-automated software for adult brain segmentation, fewer tools are available for infant brain segmentation. Similar to adult studies, manual segmentation of the infant’s brain is considered the most reliable and accurate method to identify and study brain structures (Devi et al., 2017). Manual segmentation of the brain is the “gold standard” method for segmentation (Morey et al., 2009). During the first 2 years of life, segmentation of brain MRI can be challenging due to the ongoing myelination process and frequently occurring artifacts in infant MR images due to movement (Weisenfeld et al., 2006).

Several studies have provided protocols for manual segmentation of adult MR images (e.g., Pruessner et al., 2000; Morey et al., 2009; Moore et al., 2014; Wenger et al., 2014). However, due to different contrast and the comparatively lower resolution of the infants’ brain MR images (Gousias et al., 2012), the adult protocols cannot be used directly in segmenting the infant’s brain. The resolution of the infant images, even at the standard 1 mm^3^, is comparatively worse than that for typical adult scans as the infant brain size is roughly one-third of the adult brain (Hill et al., 2010; Holland et al., 2014). Additionally, matching the resolution of the infants’ images to the resolution of the adults’ images would require an increase in total acquisition time that is not feasible. A few manual segmentation protocols have been specifically designed for segmentation of infants’ brains (e.g., Gousias et al., 2012; De Macedo Rodrigues et al., 2015; Alexander et al., 2017, 2019). However, the focus of the first study was on 15 preterm infants and only 5 term infants. The second study was done on infants who were between 0 and 2 years old, and only four of the subjects were 1–4 weeks old. The third and fourth studies replicated adult atlas in infants by manually segmenting 10 neonate MR images. Moreover, several automated methods exist for infant brain segmentation (e.g., Prastawa et al., 2005; Weisenfeld et al., 2006; Chiverton et al., 2007; Shi et al., 2010; Wang et al., 2011; Dai et al., 2012; Guo et al., 2015; Beare et al., 2016; Devi et al., 2017; Makropoulos et al., 2018; Zhu et al., 2019). A number of studies have validated manual segmentation methods for adults and compared them to automated segmentation methods; however, few studies have done this for infants. Existing studies have compared brain automated segmentation methods to manual tracing in the infant brain, but for structures other than the hippocampus and amygdala (e.g., Kempton et al., 2013; Išgum et al., 2015). To the best of our knowledge there has been no previous comparison of automated and manual segmentation of the hippocampus and amygdala in infants.

Similar to other parts of the brain, the amygdala and hippocampus start to grow and develop in the prenatal period and continue to mature into early adulthood (Uematsu et al., 2012; Thompson et al., 2013). The hippocampus is a curved structure that is located in the medial temporal lobe of the brain, beneath the cortical surface; it is one of the main structures in the limbic system. It is involved in storing long-term memory and spatial navigation among other functions (Bouix et al., 2001; Bonnici et al., 2012; Zeidman and Maguire, 2016). The amygdala is another structure in the limbic system and is closely related to the hippocampus. It is located in the temporal lobe of the brain, anterior to the hippocampus and is responsible for the perception of emotions and motivation among other functions (Hajek et al., 2009; Solano-Castiella et al., 2010; Hanson et al., 2012).

Therefore, the aim of this work was to develop a simple, easy-to-follow, and practical strategy for manual segmentation of the amygdala and hippocampus in T2-weighted infants’ brain MRI. The protocol was tested in both T1- and T2-weighted MR images. Additionally, we compared the results from manually segmented data to automated segmented data performed by iBEAT software, which is specifically designed for automated segmentation of T2-weighted infant brain. iBEAT is a freely available package running on the Linux platform; it uses advanced image processing algorithms and can perform tasks like voxel analysis and infant brain labeling (Dai et al., 2012).

## Materials and Methods

### Participants

For this study, a representative sample of 31 infants between 2 and 5 weeks of age was chosen from a larger dataset that included 175 MRI scans. Table 1 shows the distribution of the selected background characteristics. The data were obtained from self-report questionnaires filled in by the participants at gestational week 14.

**TABLE 1:** Information about the participants (*N* = 31) is reported as mean and *SD*.

Age from conception to MRI (days)	Mean: 305.80, *SD*: 8.26
Age at MRI scan (days)	Mean: 28, *SD*: 6.24
Birth weight (g)	Mean: 3639.48, *SD*: 417.81
Birth height (cm)	Mean: 50.67, *SD*: 1.51
Head circumference (cm)	Mean: 35.03, *SD*: 1.43
Ph of the umbilical artery	Mean: 7.24, *SD*: 0.073
Ph of the umbilical vein	Mean: 7.37, *SD*: 0.055
Sex	22 girls, 9 boys
Gestational age weeks 40 + (date of birth–due date)/7	Mean: 39.87, *SD*: 1.18
Maternal BMI [height/(weight/100) ^∗∗^ 2]	Mean: 25.44, *SD*: 5.005
Maternal age at birth moment (years)	Mean: 29.70, *SD*: 5.093
Medication taken by mothers	Two mothers used selective serotonin reuptake inhibitor (SSRI)/serotonin norepinephrine reuptake inhibitor (SNRI) and one used medicine that affects central nervous system
Nicotine and alcohol used by mothers	None
Father’s age at due date (mean and *SD*, years)	31.57, 4.86
Race	Caucasian, Finnish
Education divided into three classes	Low, mid 9/31 High, vocation 7/31 High 11/30 Data not available 2/31

### Image Acquisition

The infants underwent MRI scans in the Turku University Hospital at 2–5 weeks after birth (mean 17.8 days, range 12–52), counted from the estimated due date (Tuulari et al., 2017). A Siemens Magnetom Verio 3T scanner (Siemens Medical Solutions, Erlangen, Germany) was used for the imaging. Before the scan, the infants were fed to help them sleep and then swaddled into a vacuum mattress to reduce possible limb movement. The infants were scanned during natural sleep; thus, no anesthetics were used. All children were provided with double hearing protection (ear wax and ear muffs), which provided approximately 42 dB noise reduction. The duration of the whole scanning protocol was a maximum of 60 min. The family was free to discontinue the study at any point during the protocol and the scan was aborted if the baby was not soundly asleep and/or still in the scanner, or if the baby woke up in the middle of the scanning and did not fall asleep again.

The scanning protocol included Axial Dual Echo Turbo Spin Echo (TSE) sequence, where repetition time (TR) of 12,070 ms and effective echo time (TE) of 13 and 102 ms were used to produce both PD-weighted and T2-weighted images from the same acquisition. Slice thickness was 1 mm in order to acquire isotropic 1.0 × 1.0 × 1.0 mm voxels. The total number of slices was 128. A T1-weighted 3D Magnetization Prepared Rapid Acquisition Gradient Echo (MPRAGE) sequence with isotropic 1.0 × 1.0 × 1.0 mm voxels was used for anatomical imaging as well. Acquisition parameters relevant to image contrast were TR of 1900 ms, TE of 3.26 ms, inversion time (TI) of 900 ms, and flip angle of 9 degrees. All the successful brain images were evaluated by a radiologist specializing in pediatric neuroradiology. If the neuroradiologist found abnormalities in the images, the families were offered an opportunity for a child neurological examination and consultation by an experienced pediatric neurologist. The sample in the current study is free from participants with incidental findings.

### Ethics

The study was conducted in accordance with the Declaration of Helsinki. The Joint Ethics Committee of South-Western Hospital District and the University of Turku, as well as all the relevant research sites have given their approval for all parts of the present study. Parents gave written informed consent on behalf of their baby. The ethical approval number for this study is ETMK 31/180/2011.

### Image Processing

Raw MRI DICOM images were converted to Neuroimaging Informatics Technology Initiative (NIfTI) format using dcm2nii software^1^. We then rigidly co-registered individual T1- and T2-weighted volumes to one another with FSL’s flirt (6 degrees of freedom) and matched the orientation of the UNC infant template^2^ (Shi et al., 2011) in order to similarly align all the images to the same “upright” orientation. Then the NIfTI images were converted to MINC format using the MINC tools’ version 1.5.1 developed at McConnell Brain Imaging Centre, Montreal, Canada. The computer used for segmentation was iMAC OS X 10.11.6 (EI Capitan) with 4 GHz Intel Core i7 processor and with an AMD Radeon R9 M395 2048 MB graphics card.

### Manual Segmentation

Manual segmentation of the hippocampus and amygdala was performed with the developed protocol using the Display software package version 2.0 which is a part of MINC software package. For an accurate segmentation, a brush size of 0.5 mm was selected (images had a 1-mm^3^ resolution). For better visualization, brightness and contrast were adjusted and simultaneous assessment in different axial, coronal, and sagittal planes was used. In all subjects, the manual segmentation of the amygdala and hippocampus was performed in a slice-by-slice manner to carefully trace the relevant anatomical borders. Manual segmentation was performed on one hemisphere at a time. For a three-dimensional consistency of the segmentations, the images were reviewed and revised in axial, coronal, and sagittal planes. Once the segmentations were done, the delineation of the amygdala and hippocampus, in both hemispheres, was double-checked, and the necessary adjustments were made. Finally, extra segmented voxels or empty voxels had to be removed or added in order to have smooth and even segmentation. After delineating the structures, the volumes of the manually segmented amygdala and hippocampus were automatically calculated with the minc tools’ “volume_stats” function.

### Manual Segmentation of the Hippocampus

**FIGURE 1:**
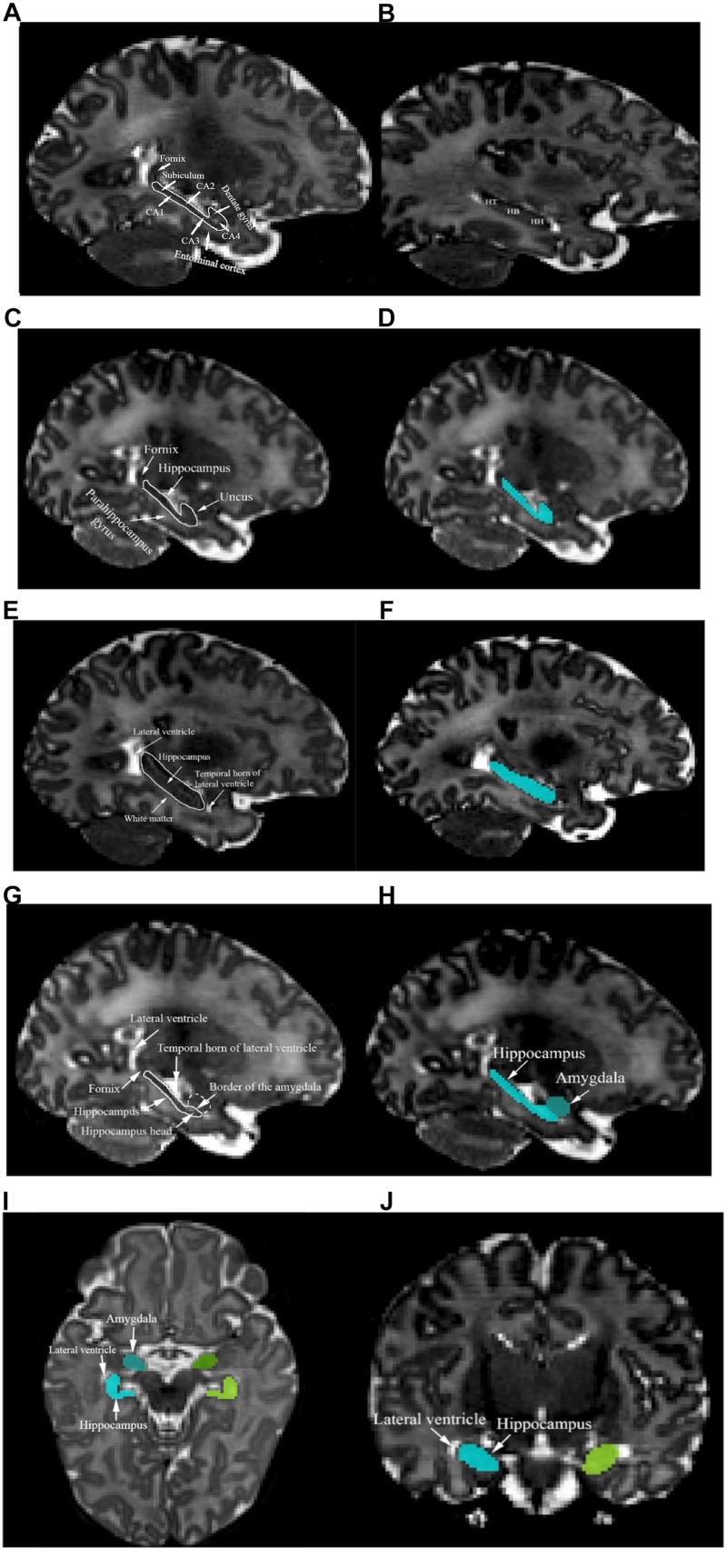
**(A)** Structures of the left fornix, entorhinal cortex, subiculum, CA1, CA2, CA3, CA4, and dentate gyrus are shown. **(B)** Left hippocampus tail (HT), hippocampus body (HB), and hippocampus head (HH) are presented. **(C)** The left fornix, parahippocampal gyrus, and uncus are presented in the sagittal plane. **(D)** The segmented left hippocampus in the sagittal plane is shown (cyan color). Left hippocampus mass was identified using landmarks such as the lateral ventricle, white matter, and the temporal horn of the lateral ventricle **(E)** and the segmented structure is shown in **(F)**. **(G)** Left hippocampus head and amygdala’s border in the sagittal view are shown. **(H)** The sagittal plane of the segmented left hippocampus and amygdala. **(I)** The axial plane of the superomedial portion of the left hippocampus (cyan), left amygdala (blue), right hippocampus (chartreuse), right amygdala (green), and lateral ventricles. **(J)** The coronal plane of the superomedial portion of the left hippocampus (cyan), right hippocampus (chartreuse), and lateral ventricle.

The hippocampus is a curved structure that is located in the medial temporal lobes of both hemispheres. With respect to the hippocampal subregions, the dentate gyrus, hippocampus proper, or cornu ammonis (CA) including CA4 region (hilus), dentate gyrus, CA3, CA2, CA1, and subiculum between CA1 and fornix were included in the segmentation as a whole (Figure 1A). The hippocampus includes three gross anatomical parts, the hippocampus head which is located in the most anterior part, hippocampus body in the medial part, and hippocampus tail in the most posterior region (Figure 1B). In this protocol, the hippocampus was defined as one region including the posterior uncus; the hooked shaped structure of hippocampus that lies at the most anterior part of the parahippocampal gyrus. The white matter track of the fimbria at the posterior portion of the hippocampus is included in the segmentation up to the point where it separates from the hippocampus and forms the fornix (De Macedo Rodrigues et al., 2015). The white matter fibers of the fornix and parahippocampal gyrus were carefully excluded from the segmentation (Figure 1C). Figure 1D shows an example of the segmented hippocampus structure.

Segmentation of the hippocampus began with identifying the borders of the most lateral hippocampal slice in the sagittal plane. Segmentation was performed on hippocampus mass, where the lateral ventricle defines the hippocampus tail and the temporal horn of the lateral ventricle appears next to hippocampus head. Moreover, the white matter appears along the hippocampus body. The inferior border of the hippocampus should be delineated, with attention paid to the contrast change between hippocampus and white matter (Figure 1E). Figure 1F represents the segmented hippocampus.

Moving inferiorly in the sagittal plane, tracing of the hippocampus mass was continued until the borders of the amygdala first became visible and the head of the hippocampus was identified using the horn of the lateral ventricle (Figure 1G). An example of the hippocampus and amygdala segmentation is shown in Figure 1H. The coronal and axial planes were used to identify the superomedial portion of the hippocampus, as the hippocampus borders are clearly distinguishable from the lateral ventricle in those planes (Figures 1I,J). When moving posteriorly in the axial view, special attention was paid to when the hippocampus and amygdala started to touch. All of the three views were referred to in order to precisely determine the borders and slices in which both the amygdala and hippocampus were present.

### Manual Segmentation of the Amygdala

**FIGURE 2:**
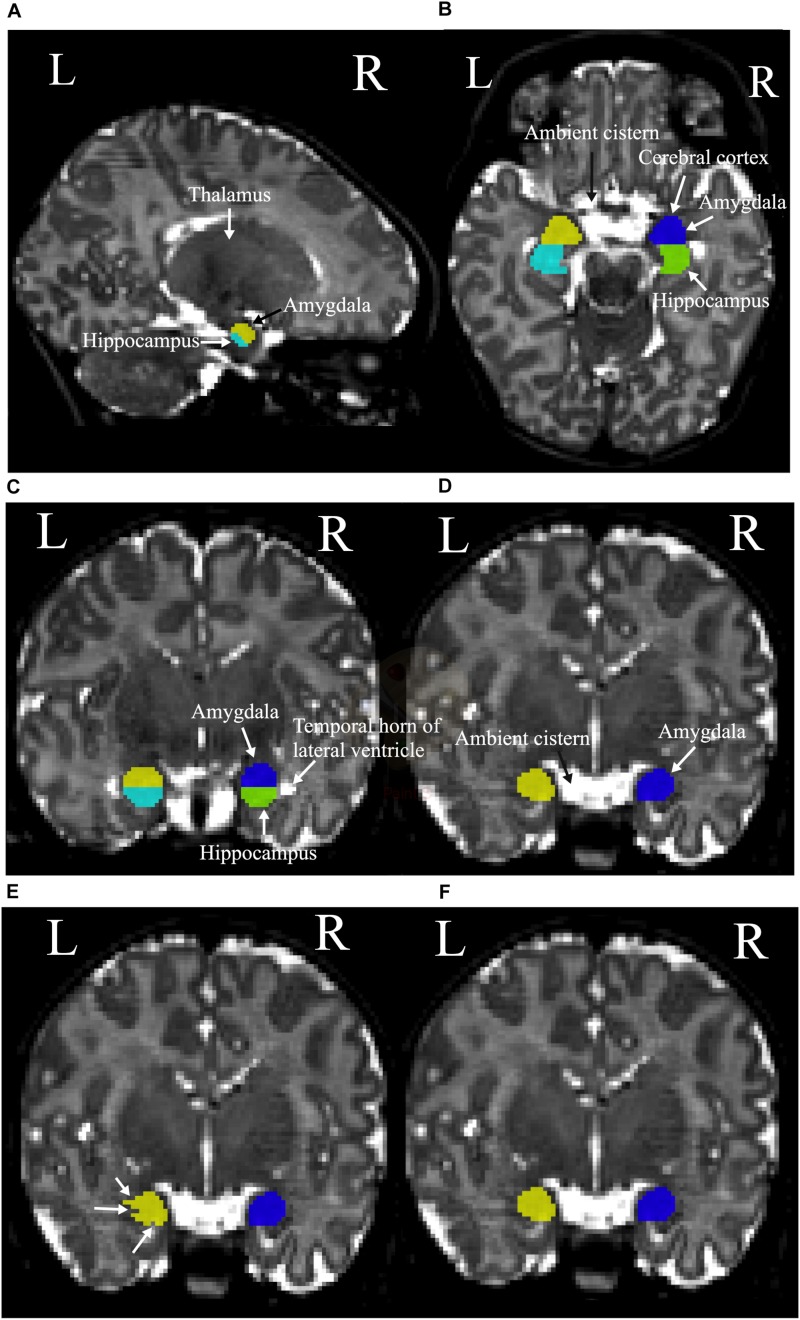
**(A)** The sagittal plane of the left thalamus, hippocampus (cyan), and amygdala (yellow). **(B)** Left and right hippocampus (cyan and green, respectively), and superior borders of the left amygdala (yellow), right amygdala (blue), ambient cistern, and cerebral cortex. **(C)** The coronal plane of the temporal horn of the lateral ventricles and left and right amygdala and hippocampus. Left amygdala with yellow, left hippocampus with cyan, right amygdala with blue, and right hippocampus with green colors are presented. **(D)** The ambient cistern, left amygdala (yellow), and right amygdala (blue) in coronal view. **(E)** Segmentation before the final adjustments is shown with white arrows. **(F)** The structure after the corrections.

The amygdala is an olive-shaped structure that is located in the medial temporal lobes of both hemispheres where it is superior and anterior to the hippocampus. Tracing the amygdala in MR images is more complicated than tracing the hippocampus, due to its location in the superomedial temporal lobe, where basal ganglia and entorhinal cortex merge into the posterior and inferior borders of this structure (Pruessner et al., 2000). As it is often not easy to identify the borders of amygdala it is important to trace the amygdala slice by slice with the help and reference to all three planes. Circa 10 slices of the amygdala were identified in each of our T2-weighted MRI data. The amygdala segmentation was started by moving superiorly in the sagittal plane from where the thalamus starts to form its walnut shape, the superomedial borders of amygdala appear superior to the hippocampus (Figure 2A). The superior border of the amygdala is attached to the ambient cistern. Therefore, it is easiest to distinguish it in the axial view. For consistency, one row of voxels at the cerebral cortex lying anterior to the amygdala was systematically excluded from delineation (Figure 2B). In the coronal plane, the temporal horn of the lateral ventricle was used to define the inferior and anterior borders of the amygdala and hippocampus, as they both were visible in the same slice (Figure 2C). Moving anteriorly in the coronal plane the lateral and inferior parts of the amygdala were identified (Figure 2D). Finally, extra segmented voxels or empty voxels had to be removed or added in order to have smooth an even segmentation (Figures 2E,F).

**FIGURE 3:**
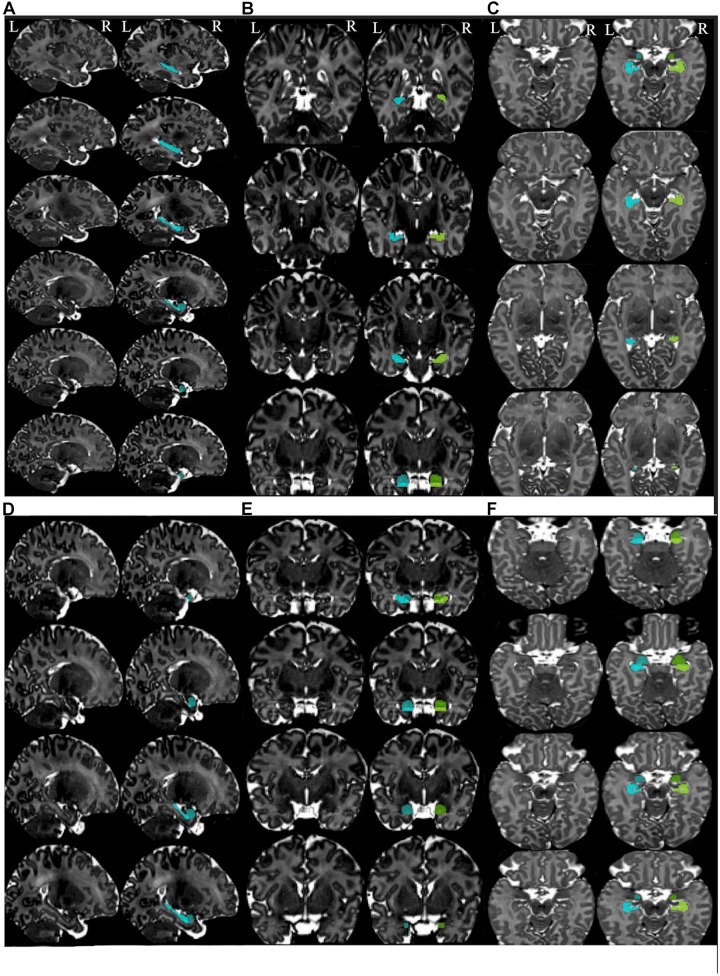
Summary of the steps for segmentation of the hippocampus and amygdala in the different planes. Summary of the steps for segmentation of hippocampus in the sagittal **(A)**, coronal **(B)**, and axial planes **(C)**. Summary of the steps for segmentation of the amygdala in the sagittal **(D)**, coronal **(E)**, and axial planes **(F)**. Left images are the templates (non-segmented) and the images at the right side present segmented structures. Left hippocampus is shown in the cyan color and the left amygdala in blue. Right hippocampus is presented in chartreuse and right amygdala in green.

The coordinates for identifying the landmarks of the hippocampus and the amygdala are presented in Table 2. MRIcron^3^ and UNC infant template (see text footnote 2) were used to specify the coordinates. It should be noted that as the MRI image of each brain varies among each individual, the coordinates are roughly applicable. A summary schematic of the performed steps for hippocampus and amygdala segmentation in sagittal, coronal, and axial planes is shown in Figures 3A–F.

**TABLE 2:** The MNI coordinates for anatomical landmarks: obtained by opening the UNC-infant-neo-withSkull infant template in MRIcron to help reproducibility.

**Structures**	***X***	***Y***	***Z***	**Hemisphere**
Hippocampus tail started to be visible	21	–26	–1	R
	–20	–24	0	L
Hippocampus head started to be visible	21	–12	–14	R
	–20	–11	–13	L
Hippocampus tail ended	13	–22	–2	R
	–13	–22	–2	L
Hippocampus head ended	13	–16	–12	R
	–11	–18	–9	L
Superior border of the amygdala started to be visible	15	–5	–8	R
	–15	–3	–7	L
Inferior border of the amygdala started to be visible	17	–3	–15	R
	–17	–3	–14	L
Superior border of the amygdala ended	20	–6	–9	R
	–20	–6	–8	L
Inferior border of the amygdala ended	21	–5	–15	R
	–20	–5	–14	L
Border of the amygdala and hippocampus	19	–9	–13	R
	–18	–9	–12	L

### Statistical Analysis

Descriptive analyses of the manual segmentation volumes were reported by mean and standard deviation (*SD*). Hemispheric volume differences between right and left amygdala and hippocampus were assessed using a paired *t*-test and *p*-values <0.05 were considered statistically significant. Data normality was checked by visual confirmation and by the Shapiro–Wilk test. The analyses were carried out using SPSS 24, Armonk, NY, United States. Additionally, Dice similarity coefficient (DSC) (Dice, 1945) was computed using Python version 2.7 in order to estimate the degree of volumetric overlap between the delineations.

We performed four different assessments: (1) intra-rater reliabilities of the main rater (NH) for baseline, 1-month intervals (*N* = 6) and 6-month (*N* = 6) intervals in between the segmentations, (2) inter-rater reliabilities between the primary rater and two less experienced raters after ca. 2 months training (*N* = 20), (3) the segmentation of the primary rater for T1- and T2-weighted images from the same participants (*N* = 10), and (4) agreement of manual tracings of the primary rater to iBEAT segmentations (*N* = 10).

### Intra-Rater Reliability Measurements

Re-segmentation was performed on 12 randomly selected MR images. Six images were segmented with 1-month intervals, and another six images with 6-month intervals. The stability of the protocol and the intra-rater reliability were assessed by comparing the segmentation volumes. Intra-rater reliability tests for the left and right amygdala and hippocampus were computed using a two-way mixed-model and absolute agreement intraclass correlation coefficient (ICC) (Shrout and Fleiss, 1979) and DSC.

### Inter-Rater Reliability Measurement

Re-segmentation was performed on 20 randomly selected MR images from the 31 images by two less experience raters using the same protocol. Each rater segmented the amygdala and hippocampus of 10 different brains. The segmentation was performed blind to the subjects’ genders and age. The volumes of the segmented structures were used to compute inter-rater reliability using a two-way mixed-model, absolute agreement, and multiple raters ICC (Shrout and Fleiss, 1979; Koo and Li, 2016). Moreover, DSC was used to report the volumetric overlap of the left and right amygdala and hippocampus between rater 1 and the main rater, and rater 2 and the main rater.

### Comparison of Manual Tracing in T1- and T2-Weighted MR Images

Manual segmentation was performed using the established protocol on 10 T1- and 10 T2-weighted images of the same subjects. Because of the ongoing myelination process and higher water content in the infant brain, the contrast in T1-weighted images is lower compared to T2-weighted images (Dubois et al., 2014; Dean et al., 2018). In the case of infants between 2 and 5 weeks of age, the intensity pattern of the white and gray matter in T1-weighted images is more similar to the adult T2-weighted images, since the white matter has lower intensity than the gray matter (Paus et al., 2001; Dean et al., 2018). Conversely, the contrast between the white and gray matter in T2-weighted images of the infants in this age group is similar to that in the T1-weighted images of adults.

By adjusting the brightness and contrast of the images, the amygdala and hippocampus are distinguishable in T1-weighted images but not as clearly as in T2-weighted images. The volumetric differences of the left and right hippocampus and amygdala in T1 and T2 images of the same subjects were extracted from the segmentations. To analyze the consistency between T1 and T2 manual segmentation, ICC and DSC were computed. T2-weighted MR images have better tissue contrast compared to T1-weighted MR images at this developmental stage. However, if the T2-weighted images are exposed to, e.g., motion artifacts, T1-weighted images could be valuable for studying different brain structures and were thus included in the protocol to study if they can be used interchangeably.

### Comparison of Automated Segmentation to Manual Tracing

Automated (using iBEAT software) and manual segmentations (using Display software) were performed on 10 T2-weighted MR images. As iBEAT software does not take infants’ T1-weighted images as input, only T2-weighted images were used for the comparison with manual segmentation. To validate the success of the automated segmentation results of the limbic structures (the hippocampus and amygdala) in iBEAT software we compared the extracted volumetric results from iBEAT to the manually defined volumes. The volumetric results from the iBEAT software and the manual segmentation were compared. Additionally, the difference between the automated segmentation results and the manual segmentation results was calculated as a percentage using Eq. 1 (Schoemaker et al., 2016):

(1) %VD=[(Va-Vm)/Vm]100*%

Using formula (1), negative percentages indicate an underestimation of the automated segmentation volumes compared to manual segmentation and positive percentages indicate an overestimation of volumes compared to manual segmentations.

Moreover, ICC and DSC were performed on the extracted volumes for the automated and manual segmentations.

## Results

### Hemispheric Difference

The mean volumes and *SD* of the left and right amygdala and hippocampus across 31 subjects are shown in Table 3. The *t-*test did not reveal significant differences between the left and right amygdala (*M*_left amygdala_ = 382.29 (mm^3^), *SD*_left amygdala_ = 124.61 and *M*_right amygdala_ = 363.96 (mm^3^), and *SD*_right amygdala_ = 110.08, *t* = 1.02, *p* = 0.31). Similarly, no significant difference was observed between hippocampus at the left and right hemispheres (*M*_left hippocampus_ = 826.38 (mm^3^), *SD*_left hippocampus_ = 109.19 and *M*_right hippocampus_ = 793.35 (mm^3^), *SD*_right hippocampus_ = 148.79, *t* = 1.64, *p* = 0.11).

**TABLE 3:** Mean and *SD* of left and right amygdala and hippocampus across 31 subjects from T2-weighted imaged done by the primary rater of the segmentation.

	**Left**	**Right**	**Left**	**Right**
	**amygdala**	**amygdala**	**hippocampus**	**hippocampus**
Mean volume (mm^3^)	382.29	363.96	826.38	793.35
Standard deviation	124.61	110.08	109.19	148.79

### Intra-Rater Reliability Results

**FIGURE 4:**
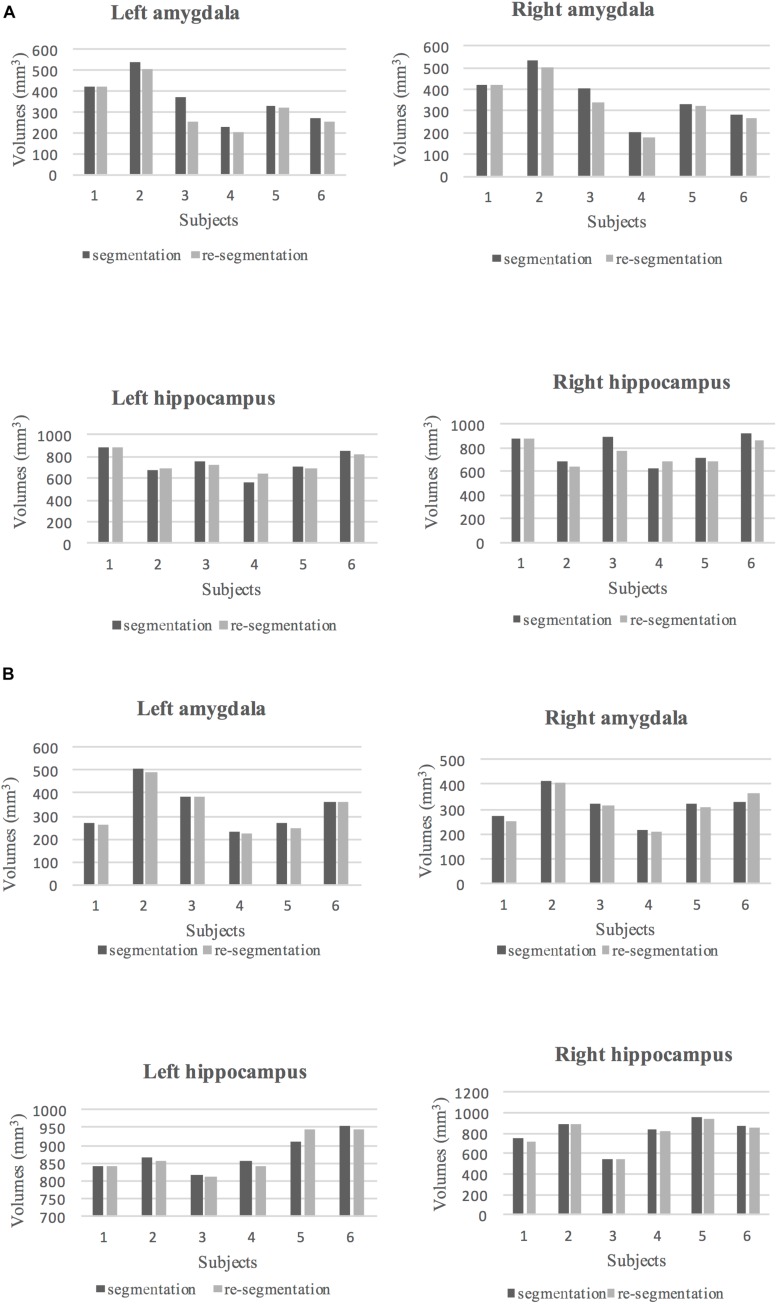
**(A)** The volumes of segmented (dark gray) and re-segmented (light gray) left and right amygdala and hippocampus for six subjects with 1-month intervals to the segmentation. **(B)** The volumes of segmented (dark gray) and re-segmented (light gray) left and right amygdala and hippocampus for six subjects with 6-month intervals to the segmentation.

To assess intra-rater reliability, ICC and DSC were calculated at two time points (at 1-month and 6-month intervals). The volumes of the segmented left and right amygdala and hippocampus at these two points are shown in Figures 4A,B. The ICC and DSC scores for intra-rater reliability with a 1-month and 6-month intervals for the left and right amygdala and hippocampus are presented in Table 4. High intra-rater reliability results were observed (ICC ≥ 0.91 and DSC ranged between 0.89 and 0.94). Thus, the manual tracings were highly replicable for a single rater.

**TABLE 4:** ICC, mean DSC, and *SD* results at two time points.

	**Left**	**Right**	**Left**	**Right**
	**amygdala**	**amygdala**	**hippocampus**	**hippocampus**
1-Month interval ICC	0.94	0.98	0.96	0.91
6-Month interval ICC	0.99	0.98	0.97	0.99
1-Month interval	0.91 (0.10)	0.92 (0.11)	0.94 (0.045)	0.94 (0.051)
DSC (*SD*)				
6-Month interval	0.89 (0.22)	0.93 (0.11)	0.91 (0.18)	0.94 (0.10)
DSC (*SD*)				

### Inter-Rater Reliability

**FIGURE 5:**
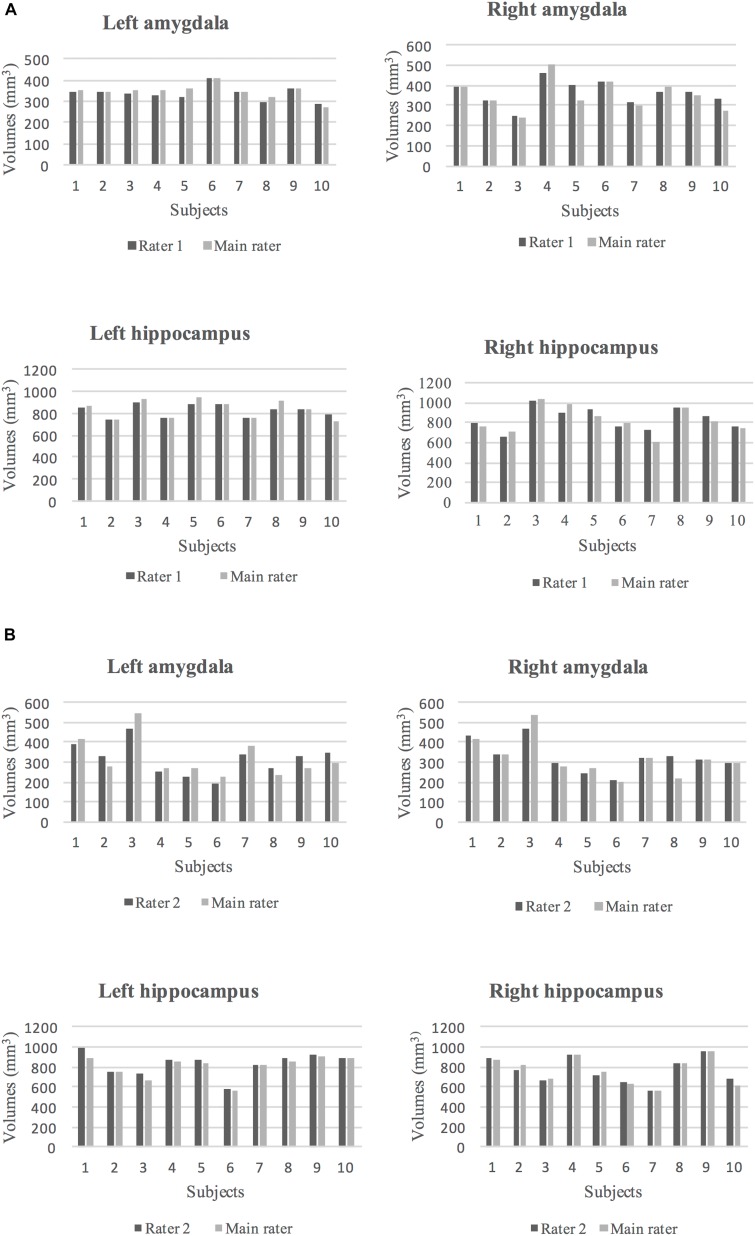
**(A)** The volumes of segmented left and right amygdala and hippocampus by rater 1 (dark gray) and the main rater (light gray) for 10 subjects are shown. **(B)** The volumes of segmented left and right amygdala and hippocampus by rater 2 (dark gray) and the main rater (light gray) for 10 subjects are shown.

The ICC and DSC results for inter-rater reliability of raters are presented in Table 5. The volumes of the segmented amygdala and hippocampus by two raters are shown in Figures 5A,B. Strong ICC and satisfactory DSC results were observed for hippocampus tracings among raters (ICC ≥ 0.90, DSC ≥ 0.75). The ICC scores for amygdala tracing were high as well (ICC ≥ 0.92). However, the DSC scores were not strong for the amygdala segmentation between the raters (DSC ≥ 0.52) and importantly, they indicated a systematic difference between the raters with regard to the placement of the regions of interest (ROIs) although the volumes show better agreement.

**TABLE 5:** ICC, mean DSC, and *SD* results of rater 1 and 2 compared to the primary rater for the amygdala and hippocampus in both hemispheres.

	**Left**	**Right**	**Left**	**Right**
	**amygdala**	**amygdala**	**hippocampus**	**hippocampus**
Rater 1 ICC	0.93	0.93	0.90	0.93
Rater 2 ICC	0.92	0.93	0.96	0.98
Rater 1 DSC (*SD*)	0.55 (0.16)	0.55 (0.22)	0.78 (0.067)	0.76 (0.086)
Rater 2 DSC (*SD*)	0.52 (0.11)	0.54 (0.14)	0.75 (0.033)	0.76 (0.048)

### Comparison of Manual Tracing in T1- and T2-Weighted MR Images

Manual segmentation of hippocampal and amygdala volumes in T1- and T2-weighted MR images of the same subjects showed slightly different volumes compared to each other. Generally, manually segmented volumes in T2 images showed lower values, likely due to better contrast at the borders. Therefore, manual segmentation in T1 images was associated with a slight overestimation of volumes for left and right amygdala and hippocampus.

ICC and DSC values for manual segmentations of the left and right amygdala and hippocampus in T1- and T2-weighted images of 10 subjects are presented in Table 6. Strong ICC and DSC scores were seen in both the amygdala and hippocampal segmentations in T1 and T2 images (ICC ≥ 0.90 and DSC ≥ 0.74).

**TABLE 6:** ICC, mean DSC, and *SD* between left and right amygdala and hippocampus in T1- and T2-weighted images.

	**Left**	**Right**	**Left**	**Right**
	**amygdala**	**amygdala**	**hippocampus**	**hippocampus**
ICC	0.95	0.97	0.97	0.90
DSC (*SD*)	0.82 (0.23)	0.80 (0.22)	0.79 (0.24)	0.74 (0.28)

### Comparison of Automated Segmentation to Manual Tracing

**FIGURE 6:**
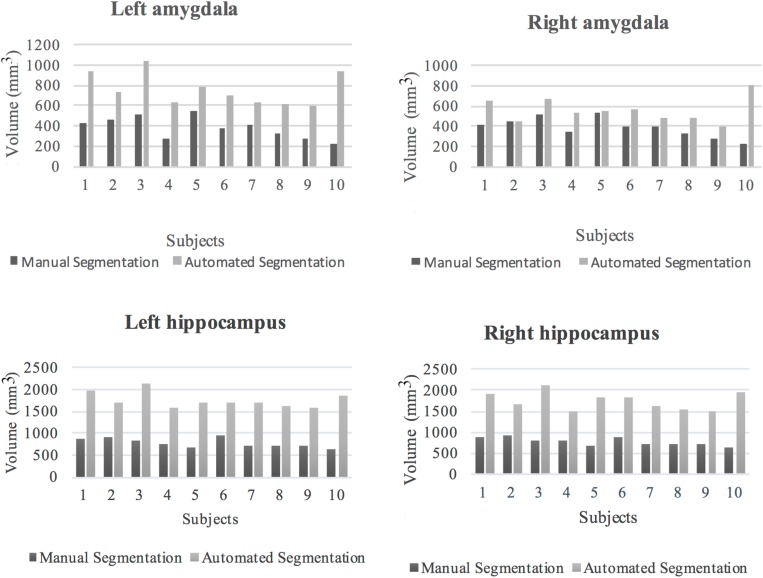
Volumes of the left and right amygdala and hippocampus from manual and automated segmentation for all 10 subjects. Volumes extracted from automated and manual segmentation are shown in light gray and dark gray, respectively.

The volumes of the manually defined structures were differential compared to iBEAT automated segmentation for both the hippocampal and amygdala segmentations. In general, automated segmentation volumes of the left and right amygdala showed greater values related to manual segmentation of those regions. Likewise, automated segmentation of the left and right hippocampus produced greater values than manual segmentation (Figure 6).

**FIGURE 7:**
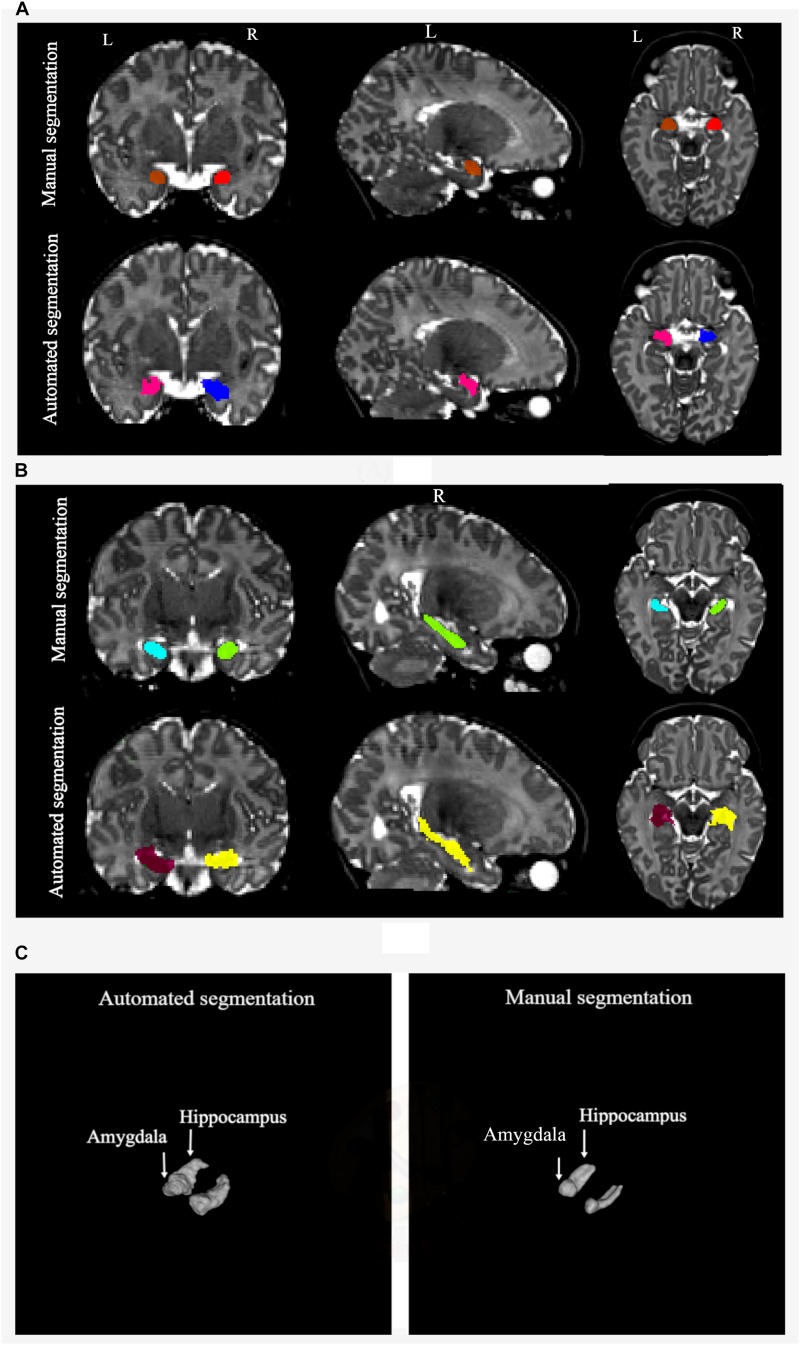
**(A)** Manual and automated segmentation of amygdala are compared. In automated segmentation performed by iBEAT, amygdala volume is overestimated. It is extended to CSF and lateral ventricles. **(B)** Manual and automated segmentation of hippocampus are compared. In automated segmentation performed by iBEAT, hippocampus volume is overestimated. It is extended to CSF, lateral ventricles, and fornix. **(C)** 3D surface render for comparing of automated and manual segmentation.

The mean percentages and SD of volume difference between the two segmentation methods for the left and right amygdala and left and right hippocampus were calculated in all the subjects (Table 7). In all of the subjects, automated segmentation overestimated volumes of the left and right hippocampus and left and right amygdala. The left and right hippocampus and left and right amygdala yielded large percentage of volume differences. The mean and standard deviation of the percentage of volume difference for the left and right amygdala were 111.8%, *SD* = 71.6 and 55.9%, *SD* = 70.5, respectively. The mean percentage of volume difference and standard deviation for the left hippocampus were 130.3%, *SD* = 32.8 and for the right hippocampus 128.4%, *SD* = 39.3. Overall, automated segmentation overestimated the volumes of amygdala and hippocampus compared to manual tracing (Figures 7A–C).

**TABLE 7:** The mean percentage of volume difference and *SD* between automated and manual tracing of the left and right amygdala and hippocampus.

Mean % of left amygdala	111.8
*SD* of left amygdala	71.6
Mean % of right amygdala	55.9
*SD* of right amygdala	70.5
Mean % of left hippocampus	130.3
*SD* of left hippocampus	32.8
Mean % of right hippocampus	128.4
*SD* of right hippocampus	39.3

ICC and DSC results between manual segmentation and automated segmentation methods for the left and right amygdala and hippocampus are presented in Table 8. No strong ICC and DSC were observed between the two methods (ICC ≥ −0.07 and DSC ≥ 0.39).

**TABLE 8:** ICC, mean DSC, and *SD* between automated and manual segmentation.

	**Left**	**Right**	**Left**	**Right**
	**amygdala**	**amygdala**	**hippocampus**	**hippocampus**
ICC	0.36	−0.07	0.21	0.08
DSC (*SD*)	0.41 (0.09)	0.46 (0.09)	0.39 (0.04)	0.40 (0.04)

## Discussion

We have developed a protocol for segmenting the amygdala and hippocampus in T2-weighted MR images of infants between 2 and 5 weeks old and confirmed that this protocol provides accurate delineations of these structures for a single rater. On the other hand, while the ICC values were satisfactory for inter-rater assessments the DSC values indicate that the labeling is not overlapping consistently; for studies using multiple raters, this is imperative to assess. We also observed a low agreement of manual tracings to automated segmentation results, but we would like to stress that we think iBEAT is a well working software for the rest of the brain (Lehtola et al., 2018), and would like to point out the importance of assessing carefully, whether a given pipeline produces the wanted and reliable outcome metrics.

Based on our experience, the overall anatomy of infants’ amygdala and hippocampus structures is quite similar to the adult brain. Nevertheless, the hippocampal folding is slightly less pronounced, and the central amygdala is frequently easier to see than in adult T1-weighted images. However, the inferior and lateral borders of both structures are more challenging to find. In this protocol, the macro-anatomical structures and boundaries were carefully included in the segmentations. Much attention has been directed to detecting and omitting the fornix and parahippocampal gyrus as parts of the hippocampus, as well as accurately identifying the boundaries of the amygdala and hippocampus adjacent to the temporal horn of the lateral ventricles. The structures and boundaries of ROIs were identified using all the three planes (sagittal, axial, and coronal); in other studies, one plane was considered as the default view and other planes were reviewed whenever needed. This protocol was tested by two raters with very little previous knowledge about manual segmentation, and they were able to quickly learn and apply the strategy employed in this protocol. High intra-rater and inter-rater reliability evaluated using intraclass correlation coefficient tests confirm that the designed protocol in this study delivers a precise step-by-step guide for the hippocampal and amygdala delineation in infant brain MRIs. Similarly, the Dice coefficient scores for intra-rater test were high. However, the DSC values for inter-rater reliability of the amygdala were not high. This is likely due to the systematic difference between the raters and small size of the amygdala and the difficulty delineating it.

Additionally, the designed protocol was used to study the variations between segmenting T1- and T2-weighted images of the same participants. Compared to T1-weighted images, manual delineations of infant T2-weighted images were easier to perform due to the better contrast at the boundaries of the structures. Segmentation of T1- and T2-weighted images provided similar, but not the same, results. Segmented volumes of the amygdala and hippocampus from T1-weighted images showed a small overestimation compared to those based on T2-weighted images. According to the strong correlation and high DSC scores between T1- and T2-weighted images, it can be concluded that T1- or T2-weighted images can be substituted for one another in the related studies (Dean et al., 2018). However, the scan type should likely be included as a covariate as the small differences may be crucial. Overall, the designed protocol offers reliable and relatively simple guidelines for segmenting the complex amygdala and hippocampal structures in infants and it is potentially useful for infant neuroimaging research projects.

We also reported the accuracy of automated segmentations of the amygdala and hippocampus performed by iBEAT software in contrast to manual segmentations of these structures. According to the calculated volume difference, percentage of volume difference, ICC, and DSC between iBEAT and manually defined structures of the amygdala and hippocampus, automatic segmentation with iBEAT was not be able to be validated against manual segmentation, which was considered the “gold standard.” iBEAT overestimated the volumes of the left and right amygdala by 111.8 and 55.9%, respectively. 130.3 and 128.4% volume overestimation were observed for the left and right hippocampus, respectively. Also, results from the correlation between manual and automated segmentation showed a high degree of disagreement between the two methods. iBEAT overestimates the amygdala segmentation by considering parts of the CSF and lateral ventricles as the structure of the amygdala. Overestimation of the hippocampus is mainly due to extending the segmentation of this structure to the CSF, fornix, and lateral ventricles. iBEAT’s overestimation of the hippocampal and amygdala volumes is likely due to the limited contrast in the infant brain, and adjacent cortical gray matter is likely being misclassified as being part of the hippocampus and amygdala.

## Conclusion

In pediatric studies, it is important to evaluate and validate automatically segmented structures. Automated approaches can be validated by using a dataset of manually segmented structures. In this study, we have described a manual segmentation protocol by which such a dataset can be produced. We hope this protocol assists the development and assessment of automated segmentation procedures of neonatal brain.

## Ethics Statement

The study was conducted in accordance with the Declaration of Helsinki. The Joint Ethics Committee of South-Western Hospital District and the University of Turku, as well as all the relevant research sites have given their approval for all parts of the present study. Parents gave a written informed consent on behalf of their baby. The ethical approval number for this study is ETMK 31/180/2011.

## Author Contributions

NH analyzed the data (preprocessing, manual and automated segmentation, and statistical analyses) and drafted the manuscript. JT supervised NH. HM and JT planned the analyses. KL and IL performed the manual segmentation. JS implemented the scanning protocol. RP planned the studies and screened the images. TL performed the clinical examinations to those with incidental findings. SL collected the data. MK and SL recruited the participants. JL planned the imaging sequences. NS was involved in planning the study and supervising the data collection. HK planned and established the cohort and provided funding for the data collection. LK co-planned and established the cohort with HK. The manuscript was revised and accepted by all co-authors.

## Conflict of Interest

The authors declare that the research was conducted in the absence of any commercial or financial relationships that could be construed as a potential conflict of interest.

## Footnotes

1 https://www.nitrc.org/projects/dcm2nii

2 https://www.nitrc.org/projects/pediatricatlas

3 https://www.nitrc.org/projects/mricron
